# The short and long-term effects of a lifestyle intervention in children with mental illnesses: a randomized controlled trial (Movementss study)

**DOI:** 10.1186/s12888-023-04884-9

**Published:** 2023-07-21

**Authors:** Emilie M. A. van Tetering, Jet B. Muskens, Jeroen Deenik, Sigrid Pillen, Wiepke Cahn, Inès von Rosenstiel, Mieke Oomen, Nanda N. Rommelse, Wouter G. Staal, Helen Klip

**Affiliations:** 1 Karakter Child and Adolescent Psychiatry, Nijmegen, The Netherlands; 2 Department of Cognitive Neuroscience, Donders Institute for Brain, Cognition and Behaviour, Radboudumc, Nijmegen, The Netherlands; 3 Department of Psychiatry, Radboud University Medical Centre, Nijmegen, The Netherlands; 4 GGz Centraal, Department of Science, Amersfoort, The Netherlands; 5 School for Mental Health and Neuroscience, Maastricht University, Maastricht, The Netherlands; 6 Kinderslaapexpert BV (Pediatric Sleep Expert Ltd), Mook, The Netherlands; 7 Department of Psychiatry, University Medical Centre Utrecht, Utrecht, The Netherlands; 8 Department of Pediatrics, Rijnstate Hospital, Arnhem, The Netherlands; 9 GGz Eindhoven, Eindhoven, The Netherlands; 10 Leiden Institution for Brain and Cognition, Leiden, The Netherlands

**Keywords:** Lifestyle intervention, Mental illness, Children, Treatment, Short and long-term effects

## Abstract

**Background:**

A lifestyle including poor diet, physical inactivity, excessive gaming and inadequate sleep hygiene is frequently seen among Dutch children. These lifestyle behaviors can cause long-term health problems later in life. Unhealthy lifestyle and poor physical health are even more prevalent among children with mental illness (MI) such as autism, attention-deficit/hyperactivity disorder, depression, and anxiety. However, research on lifestyle interventions among children with MI is lacking. As a result, there are currently no guidelines, or treatment programs where children with MI and poor lifestyle can receive effective support. To address these issues and to provide insight into the effectiveness of lifestyle interventions in children with MI and their families, the Movementss study was designed. This paper describes the rationale, study design, and methods of an ongoing randomized controlled trial (RCT) comparing the short-term (12 weeks) and long-term (1 year) effects of a lifestyle intervention with care as usual (CAU) in children with MI and an unhealthy lifestyle.

**Methods:**

A total of 80 children (6–12 years) with MI according to DSM-V and an unhealthy lifestyle are randomized to the lifestyle intervention group or CAU at a specialized child and adolescent mental hospital. The primary outcome measure is quality of life measured with the KIDSCREEN. Secondary outcomes include emotional and behavior symptoms, lifestyle parameters regarding diet, physical activity, sleep, and screen time, cognitive assessment (intelligence and executive functions), physical measurements (e.g., BMI), parenting styles, and family functioning, prior beliefs, adherence, satisfaction, and cost-effectiveness. Assessments will take place at the start of the study (T0), after 12 weeks (T1), six months (T2), and 12 months of baseline (T3) to measure long-term effects.

**Discussion:**

This RCT will likely contribute to the currently lacking knowledge on lifestyle interventions in children with MI.

**Trial registration:**

trialsearch.who.int/ NL9822. Registered at November 2^nd^, 2021.

## Background

In recent years, increasing attention has been given to lifestyle and their effects on mental health. Various studies consistently show that adults with mental illness (MI) have higher rates of physical ill health and die earlier than those in the general population, largely due to treatable conditions associated with modifiable risk factors such as smoking, obesity, substance misuse, and adverse effects of medication [1, 2]. A recent meta-analysis shows promising effects of lifestyle interventions in adults with MI on their mental health [3].

Unhealthy lifestyle and poor physical health are also frequently seen among children with MI, such as autism spectrum disorders (ASD), Attention Deficit Hyperactivity Disorder (ADHD), depression, and anxiety disorders, compared to children without MI [4, 5]. An unhealthy lifestyle among children consists, for example, of low physical activity [6], poor nutrition intake [7], disrupted sleep [8, 9], and elevated screen time [5, 10]. An unhealthy lifestyle in the family may have a major impact on the mental, physical and social functioning of children [11]. However, longitudinal studies of lifestyle interventions in children with MI are relatively sparse [3].

Research on lifestyle-related factors and integrated lifestyle programs for children and their families in the general population is increasing [12, 13]. Nevertheless, children with MI are generally excluded from these integrated lifestyle programs because of the perceived complexity of care for these families. Therefore, data on lifestyle interventions for children with MI and their caregivers are sparse. Moreover, treatment expertise of an integrated approach to lifestyle factors in child mental health hospitals is lacking. A major concern is that children with MI are at high risk of developing chronic physical and mental health issues and, consequently, require lifelong healthcare services because of the complex interaction between unhealthy lifestyle, physical and mental comorbidities, and adverse effects of psychotropic medication at a young age [3]. The main lesson from other lifestyle interventions, such as those developed for obesity, is that the later the start of intervention for physical comorbidities, the poorer the outcomes at an older age [14]. From a preventive perspective, a multimodal lifestyle intervention program for children with MI is needed to improve mental and physical outcomes.

Based on the programs for children without MI, the Movementss study has been developed. This is a multi-modal lifestyle intervention program with a systematic approach focusing on four lifestyle parameters (i.e., movement/physical activity, nutrition intake, sleep behavior, and screen time restoration) for children with MI and their families. The trial will focus on the relationship between an integrated lifestyle intervention program and the quality of life for children with mental illness and their families. To assess the added value of this lifestyle intervention, children will be randomized into two groups: intervention and care as usual group. The intervention involves family-based education on a healthy lifestyle in combination with cognitive behavior therapy (CBT) for the child and psychomotor systematic therapy (PMST) for the whole family given by mental health professionals. In PMST, the child and caregiver work in a body-oriented manner, and they both learn concrete skills (i.e., an action-oriented approach) [15–17]. Also, during PMST, systemic interaction patterns between family members will be established. Furthermore, from integrated lifestyle intervention programs for children without mental illnesses, it is known that children need support from their families, and unhealthy lifestyle habits need to be targeted using a family-based intervention [18]. For this reason, caregivers and mental health professionals will actively participate in the intervention. To generalize healthy behavior in the family, a home coach will be involved to visit the child and family at their homes.

Since a healthy lifestyle contains multiple elements (i.e., nutrition, sleep, screen time, and physical activity), the intervention will have a tailored approach, where the child and caregiver will take the lead in their goal setting. An integrated approach focusing on multiple lifestyle factors (including psycho-education and skills training), social (peer)support, and guided by qualified professionals does have the best chance of success [19–21]. For lifestyle interventions to succeed, it is highly relevant that the focus on lifestyle factors and specific goals is tailored to the needs, interests, and environment of the participants [19]. Using goal setting in this integrated and tailor-made intervention allows for easier translation to daily life.

The primary objective of the Movementss study is to quantify the short-term (after 12 weeks) and long-term (after 6 and 12 months) effectiveness on quality of life compared to care as usual (CAU) in children aged 6–12 years with MI. We hypothesize that the lifestyle intervention will be more effective than CAU. The second aim is to examine the short- and long-term effects of the lifestyle intervention compared to CAU on outcome measures related to mental and physical symptoms and lifestyle problems such as emotional and behavioral problems, cognitive outcomes, sleeping, eating patterns, and weight status. It is hypothesized that lifestyle improvement will have a positive effect on these mental and physical symptoms. Additionally, the third aim is to investigate the cost-effectiveness of the lifestyle intervention compared with CAU. We assume that intervening in lifestyle at such a young age will result in less expensive care in the long term. Also, this study aims to identify potential moderators of adherence or responders to the treatment (e.g., SES, ethnicity, comorbidity). Finally, qualitative questions will be addressed in this study by examining the potential facilitators and barriers to the lifestyle intervention.

## Method

### Study design

This study uses a mixed-methods design combining a two-arm randomized controlled trial with semi-structured interviews to capture caregivers’ and professionals’ experiences before and after the lifestyle intervention, using repeated measures and semi-structured interviews. The aim of combining quantitative and qualitative components within this study is to provide a better understanding of the effectiveness of the lifestyle intervention.

### Quantitative study

#### Participants

The medical ethics Committee on Research involving Human Subjects of Radboudumc (CCMO, Arnhem-Nijmegen) approved the study and it is registered under 2021–8224. Written informed consent regarding participation and publication from all parents or guardians for participants under 16 years will be obtained during the randomized controlled trial.

Participants will be recruited among new referrals at Karakter Child- and Adolescent Psychiatry and Denkkracht, The Netherlands. Karakter is a hospital for specialist mental health care, and Denkkracht a center for neuropsychological expertise. The selection of participants is a 2-step process. First, our digital intake questionnaire is used to select potential participants. Before the first appointment at the clinic, parents must complete this digital intake questionnaire. This assessment includes age, sex, country of birth, and parents’ educational level. In the section on lifestyle, various questions about physical exercise, healthy eating behavior, sleeping patterns, and screen time are asked. Potential participants are selected when they positively respond to one or more of the following questions:

1. How much time does your child spend in front of a screen compared to peers (*More* or *Much more*)
2. How much exercise does your child have compared to peers? (*Less* or *Much less*)
3. How often does it take longer than 30 min for your child to fall asleep? (*Always*)
4. How often does your child wake up more than three times per night, or does your child lie awake for more than 20 min per night? (*Always*)
5. How often does your child wake up before 6 am? (*Always*)
6. How often does your child experience daytime fatigue? (*Always*)
7. How many days a week does your child eat breakfast? (*(Almost) never* or *1–2 days a week*)
8. How many days a week does your child eat fruit? (*(Almost) never* or *1–2 days a week*)
9. How many days a week does your child eat vegetables? (*(Almost) never* or *1–2 days a week*)
10. How many times a day does your child get snacks (biscuits, candy, chips)? (*5–6 times a day* or *More than seven times a day*)
11. How many days a week does your child eat “fast food” (pizza, chips, pancakes, chip shop, snack bar, etc.)? (*5–6 days a week* or *Every day*)

Secondly, following a positive response to these items, participants are invited for a somatic assessment and eligibility check for the Movementss study by a nurse specialist and child and adolescent psychiatrist. Somatic assessment includes measurement of weight, height, blood pressure, heart rate, head circumstances, an inspection of dysmorphic features, and motor functioning and coordination.

For eligibility, participants must meet the following criteria: 1) 6–12 years old at the inclusion date, 2) DSM-5 diagnosis (any presentation), 3) somatic or lifestyle problems assessed by somatic examination and lifestyle screening (overweight, obesity, underweight, unhealthy diet, sleeping problems, inactivity, and screen time use), and 4) willingness to set lifestyle goals. Comorbidities are allowed except for eating disorders (i.e., anorexia or bulimia nervosa) and diabetes mellitus. Exclusion criteria are: 1) unable to respond to questions (parents or children), 2) no access to a home internet connection, 3) insufficient mastery of the Dutch language in parents or children, 4) physically incapable of doing physical exercises, 5) surgery in the past six months or next twelve months impacting physical activity or dietary intake, 6) any somatic condition severely restricting diet, current treatment for the psychiatric diagnosis, and 7) an intelligence quotient (IQ) below 70. Participants receiving this lifestyle intervention cannot start any other treatment during the 12-week-long lifestyle intervention, except for the optimization of pharmacotherapy. For children who receive medication, this will be optimized during the lifestyle intervention. During this optimization, the physical health of the children will be closely monitored since lifestyle-related problems (e.g. sleeping problems, reduced appetite, and overweight) more often occur when using medication. Similarly, participants receiving CAU will receive one session of digital psycho-education on a healthy lifestyle though they will not follow a lifestyle intervention.

#### Randomization

The Movementss study is a two-arm randomized controlled trial; patients fulfilling eligibility criteria and willing to participate will be randomized after baseline assessment (1:1 allocation ratio) in either the lifestyle intervention or CAU group using block randomization, with varying random block sizes of 2, 4 or 6. A priori, an independent statistician will prepare a computer-generated randomization schedule stratified for weight (underweight, normal, overweight), sex (male, female), and age (6–8 years, 9–12 years) to ensure that the number of participants in both treatment arms is closely balanced within each stratum. Castor EDC will be used for the actual randomization process. After obtaining informed consent and baseline measurements, the study coordinator will provide the randomization code. Participants, researchers, and practitioners are aware of treatment allocations.

#### Procedures

Families interested in participating in the Movementss study are informed by telephone and information letters (sent via e-mail). If the eligibility criteria are met, parents and children are invited for an intake consult (via e-health or at the psychiatry clinic), explaining further details of participating in the study. Both parents fill out written informed consent for the Movementss study (Additional file 1).

**Fig. 1:**
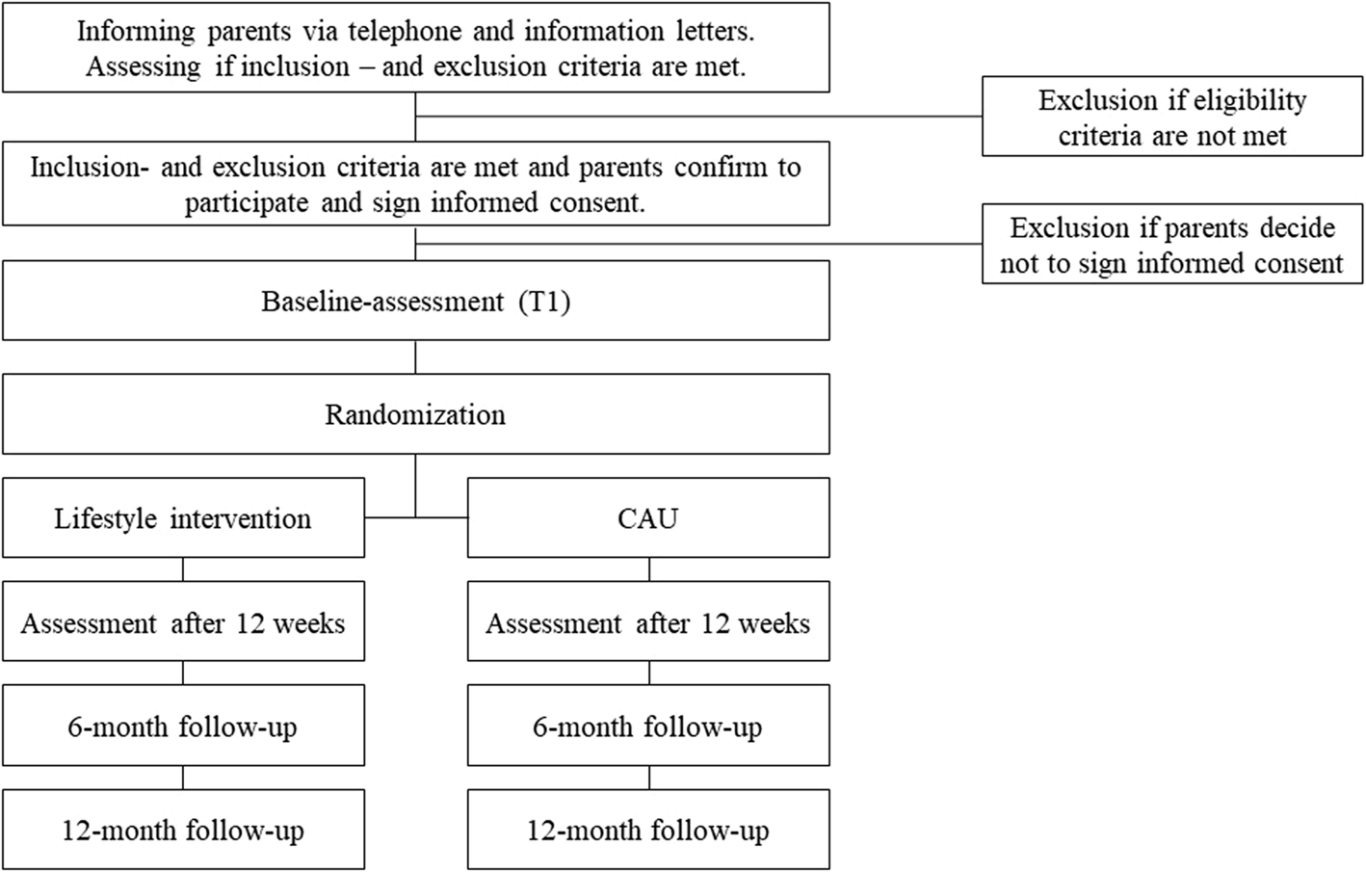
Recruitment and procedure of the study

Assessments will take place at baseline (T0), after twelve weeks of treatment (T1), after six months from baseline (T2), and twelve months from baseline (T3) (i.e., lifestyle intervention with CAU). T0 will be scheduled three weeks before the start of the treatment. T0 and T1 will take place at Karakter Child and Youth Psychiatry. For the T2 and T3 assessments, parents are asked to fill out questionnaires online. Figure 1 describes a flowchart of recruitment and the procedure for the study. It is estimated that the study’s data collection will be completed in spring 2024 (*N* = 80), and the follow-up data will be completed at the beginning of 2025.

### Interventions

#### Lifestyle intervention

The lifestyle intervention combines psycho-education on a healthy lifestyle with a lifestyle program focusing on improving physical exercise, healthy eating behaviors, sleeping patterns, and screen time. Based on previous research, an integrated approach (including psycho-education and skills training) has the best chance of being successful [19–21]. At the start of the intervention, children and their parents will set the goals they want to obtain at the end of the intervention (i.e., after 12 weeks). This is a tailored-based intervention, meaning that the goals and the resulting focus of the content of the intervention can differ for each family. However, the same techniques, structure, and working elements are used equally (see Table 1).

**Table 1:** Sessions of the lifestyle treatment at location

Session	Topic	Goal
1	Introduction to treatment	• In this session, treatment goals are established. To achieve these goals, both the child and the parent(s) must support them. Together with the practitioner, it is discussed which goals are set up for achievement in the next 12 weeks
2	Psycho-education on healthy eating behavior and exercise	• This session explains more about healthy eating behavior and physical activity
		• For healthy eating behavior, the psycho-education is based on the most recent Dutch dietary guideline and the recommended daily consumption of food groups per sex and age group developed by The Netherlands Nutrition Center (Voedingscentrum). For physical activity, parents and children will be educated using ‘the Beweegrichtlijn 2017 for children^a^
		• During the session, it is discussed how parents and children’s eating habits and exercise levels can be embedded in their lives
		• At the end of the session, the completed food diary of the child is discussed to get a better insight into their eating patterns
3	Psych-education on healthy sleeping habits and screen time use	• This session explains more about healthy sleeping habits and screen time use
		• During this session, it is discussed how the sleeping habits and amount of screen time used by parents and the child are embedded in their lives
		• At the end of the session, the completed sleeping diary of the child is discussed to get a better insight into their sleeping pattern
4	PMST: Interaction patterns and qualities	• This first PMST session aims to get a deeper insight into the different patterns in the family. Moreover, multiple assignments are done together with the whole family to get a better understanding of everyone’s role in the family. This allows interaction patterns between family members to be better investigated and understood
5	CBT: Learning about thinking	• This session aims to give a better understanding of the coherence between thoughts and behavior based on the CBT perspective. Hereby, lifestyle behavior is linked to possible underlying thoughts
6	CBT: Emotions and signaling plan	• This session aims to get a better insight into the different emotions people have. In this session, these different emotions are linked to lifestyle behaviors (e.g. happiness and cycling, or loneliness and gaming)
7	PMST: Emotions and signaling plan	• This PMST session aims to transfer what was learned from session 5 into body-oriented exercises in the gym
8	CBT: Helping thoughts. PMST: feedback on video’s from PMST together with parents	• This session aims to understand ‘helping thoughts’. Thoughts can help when children are worried about things. These helping thoughts are linked to situations when it is difficult to maintain a healthy lifestyle behavior and the obtained goals
		• In the same week, parents look back at the video recordings from PMST sessions. The therapist provides feedback on these sessions with the aim of gaining a better understanding of the interactions in the family
9	PMST: Experimenting with new behavior	• This PMST session aims to practice new behavior linked to the goals. Together with the PMT therapist assignments on goal-setting are done body-oriented
10	PMST: Positive attention and ‘breaking the negative interaction spiral’	• This PMST session aims to get a better insight into the interaction patterns in the family. The focus lies on increasing positive attention and breaking the negative interaction spiral
11	Relapse prevention	• In this session, the learned behavior will be retained. Furthermore, a joint plan is made with steps to take when maintaining the new behavior becomes difficult again (i.e., a relapse prevention plan)
12	Evaluation and closing	• This last session evaluates the therapy session and the goals

Parallel to these sessions on location, the family will receive visits from the homecoach. The order of the sessions is the guideline for the intervention, however, a certain degree of flexibility is required and accepted in order to make the intervention fit to the family

*CBT* Cognitive Behavioral Therapy, *PMST* Psychomotor Systematic Therapy

^a^The Beweegrichtlijn 2017 advises children (4–18 years) to exercise moderately for at least an hour a day. Longer, more frequent, and/or more intensive exercise provides additional health benefits. Furthermore, children are advised to do muscle and bone-strengthening activities at least three times per week and to avoid a sedentary lifestyle

This intervention will be given by trained mental health professionals (i.e., psychologists and nursing specialists). Prior to the start of the intervention, these professionals received a three-day training by experts in the field focusing on sleep-promoting behavior, healthy eating behavior, screen time (addiction), and motivational interviewing.

The lifestyle intervention consists of a 12-week program (see Table 1) where the child and parents are treated intensively (approximately two hours a week). The intervention will start with psychoeducation sessions on a healthy lifestyle. In addition to providing more information about a healthy lifestyle, psychological treatment is necessary to achieve and maintain behavioral change. CBT has great evidence as an effective intervention method for behavioral change in mental health problems in adults and children (e.g., sleeping problems and eating disorders) [22]. Due to the amount of evidence of CBT, and the focus on both cognitions and behavior in lifestyle behavior, this psychological treatment was chosen for this intervention. A workbook is available for the child and the parents and contains information about a healthy lifestyle and describes the various assignments in detail. See Table 1 for a more detailed description of the content of the sessions. While the order of the sessions is the guideline of the intervention, a certain degree of flexibility is needed to fit the family's needs.

These CBT sessions will be combined with PMST sessions for children and their parents. Because of this combination, children and parents can use the behavioral skills they have learned during CBT (e.g., behavioral experiments; using helpful thoughts) in assignments of the PMST. In this way, parents and children will get concrete examples of applying the learned CBT techniques. In PMST, children and their caregivers both learn skills (i.e., action-oriented approach) but also experience and get more knowledge about emotion regulation and how it feels in your body (i.e., experience-oriented approach) [16]. Furthermore, systemic interaction patterns between family members are quickly established. The PMST sessions will be held in the gymnasium.

In addition to the CBT and PMST sessions on location, there will be weekly visits by a home coach since family support at home is crucial for the child. The home coach will visit families in their homes each week to promote healthy behavior and help families apply new lifestyle skills. This home coach may also visit schools and sports clubs with the child to involve (and, if necessary, educate) teachers and trainers. The coach plays a crucial role in creating a healthy eating, exercise, and sleep environment in the family as well as setting boundaries and focusing on self-empowering the system. Targeting a child’s whole system and social context will promote lasting behavioral changes and family satisfaction. The home coach will discuss personal goals continuously with the children and their caregivers. The step from ‘no change’ to ‘some change’ is the most crucial. Targeting the family will stimulate the child and promote lasting behavioral changes.

The main focus of the treatment, regarding the different lifestyle items, is described below.

A. Sleep
  For sleep problems, the focus will be: education on sleep hygiene, addressing a shift in the biological clock, and CBT. This entails 1) psycho-education regarding normal sleep and good sleep habits; 2) choosing optimal and regular bedtimes, bedtime fading, and sleep restriction; 3) optimizing sleep hygiene, especially concerning the use of screen time before bedtime and incorporating a good ritual before bedtime; 4) optimizing the amount and timing of physical activity and light exposure; and 5) optimizing stimulant medication (optional) for the child regarding sleep problems.
B. Screen time use
  Education on a healthy lifestyle and CBT will be applied to screen time use. This entails 1) psycho-education regarding everyday screen time use, 2) choosing optimal screen use time, and 3) supporting other activities that do not include screen time.
C. Healthy eating behaviors
  For healthy eating behavior, education on a healthy lifestyle and CBT will be applied. With this, the focus will be on improving nutritional food intake. Parents and children will be educated on the most recent dietary guidelines by The Netherlands Nutrition Center, part of The European Public Health Nutrition Alliance (EPHNA) and the recommended daily consumption of food groups per sex and age group. Moreover, with parents and children, ways to make healthy food intake more accessible are investigated.
D. Physical activity
  Education on a healthy lifestyle and CBT will be applied to physical activity. The focus will lay on the improvement of the amount of physical activity. As part of the lifestyle intervention, the child will practice in the gym with a mental health professional and together with the family during PMST. Parents and children will be educated on ‘The Dutch Beweegrichtlijn 2017 for children’ which is comparable for children with the World Health Organization [23] guidelines on physical activity and sedentary behaviour to create more awareness and knowledge on the recommended amount of physical activity per week. Moreover, the therapy will focus on ways to promote physical activity and make it easier for them in daily practice.

#### Care as usual

Care as usual (CAU) consists of a 1.5 h-session of psycho-education on healthy lifestyle for the parents only. This psycho-education consult is given by a psychologist which is trained for this research. The children have to follow an e-health psycho-education module about healthy lifestyle on a device of their choice (e.g., a mobile phone, laptop, or tablet), which will take them about two hours (in total) to complete. However, this module does not have to be taken through in one go. As well as for the psycho-education consult of parents as for the e-health module of the children, the content consist of information on healthy eating behavior, healthy use of screen time, healthy sleep patterns, and physical exercise. A conscious decision was made to include psycho-education about a healthy lifestyle in the control condition. We decided to offer parents and children in the CAU group a psycho-education session on healthy living and access to the e-health psycho-education module in exchange for the information we gather during assessments. After this, the child and family will receive care as usual (e.g., optimization of pharmacotherapy).

### Outcomes

Outcome parameters and measurement times are assessed as shown in Table 2.

**Table 2:** Outcome parameters from baseline to 12 months after baseline

Measurement	T0^a^	T1^b^	T2^c^	T3^d^	Instrument
*Descriptives*					
A. Descriptives	T0				Intake questionnaire
B. Psychiatric evaluation	T0				DSM-V
C. Intelligence quotient (IQ)	T0				WISC-V
*Primary outcome*					
D. Quality of life	T0	T1	T2	T3	Kidscreen-27
*Secondary outcomes*					
E. Emotional and behavior problems	T0	T1	T2	T3	CBCL
F. Cognitive performance	T0	T1			COTAPP
G. Somatic measurements	T0	T1		T3	Somatic screening
H. Blood laboratory tests	T0				Blood samples (15 ml) sober
*Lifestyle parameters*					
I. Sleep: Sleeping habits	T0	T1	T2	T3	CSHQ
J. Sleep: Sleeping dairy	T0	T1	T2	T3	Sleep–wake diary
K. Sleep: 24 h sleep/wake measurements	T0	T1	T2	T3	Actigraphy
L. Diet: Food consumption	T0	T1	T2	T3	Nutrition diary
M. Diet: Eating habits	T0	T1	T2	T3	Eating habits questionnaire
N. Physical activity: Physical fitness	T0	T1		T3	Shuttle run test
O. Physical activity: Physical activity	T0	T1	T2	T3	Physical activity questionnaire
K. Physical activity: Actigraphy	T0	T1	T2	T3	Actigraphy
P. Screen time: Screen activities	T0	T1	T2	T3	Screen time questionnaire
*Measurements of costs*					
Q. Used healthcare resources				T3	Tic-P questionnaire
*Other study parameters*					
R. Parenting styles	T0	T1	T2	T3	VSOG
S. Parenting functioning	T0	T1	T2	T3	VGFO
T. Prior beliefs	T0				5-item questionnaire
U. Patient satisfaction			T2	T3	GGZ-Jeugdthermometer

*DSM* Diagnostic and Statistical Manual of Mental Disorders, *WISC* Wechsler Intelligence Scale for Children, *IQ* Intelligence quotient, *CBCL* Child Behavior Checklist (6–18), *CSHQ* Children’s sleep habit questionnaire, *Tic-P* Trimbos and iMTA questionnaire on Cost associated with Psychiatric illness, *VSOG* Verkorte Schaal voor Ouderlijk Gedrag, *VGFO* Vragenlijst Gezinsfunctioneren Ouders

^a^T0 Baseline

^b^T1 twelve weeks after baseline

^c^T2 six months after baseline

^d^T3 twelve months after baseline

#### Descriptives

- A. Descriptives
  The children’s primary caregivers are asked to report personal characteristics for themselves (gender, age, country of birth, highest completed education, family composition, living situation, and occupational status), their partner - if any (gender, age, country of birth, most increased completed education and occupational status)- and the child (country of birth, medical history, and school parameters).
- B. Psychiatric evaluation
  Based on the Diagnostic and Statistical Manual of Mental Disorders, Fifth Edition (DSM-5) [24] the psychiatric diagnosis is assessed by a multidisciplinary team consisting of child and adolescent psychiatrists, psychologists, and nurse practitioners. For the descriptives, a primary diagnosis is presented. Other diagnoses are grouped as co-morbidity. This is standard practice at Karakter Child and Adolescent psychiatry.
- C. Intelligence quotient
  Total IQ is estimated using the Wechsler Intelligence Scale for Children (WISC-5), the best-known and most widely used intelligence test in the Netherlands for children [25]. The WISC-V is suitable for children from 6 to 16 years. WISC-V will only be administered if it hasn't been taken in the past two years. Otherwise, the outcomes from a previous WISC-V are used. IQ is assessed only at T0 (Baseline).

#### Primary outcome

- D. Quality of life
  Parents are invited to rate the children’s quality of life using the KIDSCREEN-27 [26]. The KIDSCREEN-27 is a generic health-related quality of life (HRQOL) questionnaire for children and adolescents applicable for healthy and chronically ill children and adolescents aged between 8 and 18 years [26]. The KIDSCREEN-27 consists of 27 items that measure five dimensions: physical well-being, psychological well-being, parent relations & autonomy, social support & peers, and school environment. Items are answered on a five-point Likert-type scale assessing frequency: *never* (1), *seldom* (2), *sometimes* (3), *often* (4), and *always* (5), or intensity: *not at all* (1), *slightly* (2), *moderately* (3), *very* (4), and *extremely* (5), with a 1-week recall period. Scores are coded from 1 to 5, negatively formulated items will be recoded, and the sum scores for respective dimensions are transformed to* T* scores with a mean of 50 and a standard deviation (SD) of 10: higher scores indicate better HRQOL [26]. The KIDSCREEN-27 has been shown to have robust psychometric properties [26]. The internal consistency of the domains was between 0.81 and 0.84, and the test-retest reliability of the domains ranged from 0.61 to 0.74 [26]. Quality of life is assessed at T0 (Baseline), T1 (end of intervention), T2 (six months after baseline), and T3 (12 months after baseline).

#### Secondary outcomes

- E. Emotional and behavioral problems
  The Dutch parent-report version of the Child Behavior Checklist 6–18 years (CBCL) assesses a wide range of children's emotional and behavioral problems, aiming to identify children at high risk of a psychiatric disorder [5]. The CBCL/6–18 comprises 120 items assessing behavioral and emotional problems that are answered on a 3-point Likert-type scale (0 = *not true,* 1 = *somewhat or sometimes true,* 2 = *very true or often true*) by parents [5]. The scores display eight problem scales: withdrawn (1); somatic (2); anxious (3); social (4); thought (5); attention (6); rule-breaking (7); aggressive (8); and other problems, the sum of the problem scale 1,2 and 3 form the scale ‘internalizing behavior’; 7 and 8 form ‘externalizing behavior’ [5]. All subscales together count for the total problem scale [5]. Some items contribute to more than one problem scale. T scores are computed from raw scores; higher scores on the syndrome scales indicate greater severity of problems. A T score of 63 (90th percentile) demarcates the clinical range, indicating that a child needs professional help [5]. The CBCL/6–18 has well-established psychometric properties in clinical, non-clinical, and cross-cultural populations [27]. The CBCL is assessed at T0 (Baseline), T1 (end of intervention), T2 (six months after baseline), and T3 (12 months after baseline).
- F. Cognitive performance
  The Cognitive Task Application (COTAPP) measures cognitive performance [28]. As described by Bosch, Bierens [29], this Dutch neurocognitive assessment tool is designed to examine (variability in) processing speed, attentional and executive control, working memory, and learning speed with a computer task. The COTAPP is a two-choice reaction time paradigm with seven blocks, in which the child is assisted through the different paradigms in a playlike way [28, 29]. By default, no coaching or help is given to the child. If the child fails in completing the task without the assistance of the examiner, coaching or help can be provided in a structured manner [28, 29]. The amount of offered coaching or help, is coded by the examiner and included in the outcome parameters [28, 29]. In addition, the level of verbal and motor activity of the child during performance can be coded. Validity and reliability of the COTAPP have been confirmed [28], with COTAPP relating significantly to intelligence, school performance, ADHD and Autism Spectrum Disorder symptoms, and the quality of the student-teacher relationship [29]. The COTAP is assessed at T0 (Baseline) and T1 (end of intervention).
- G. Somatic measurements
  Physical examination includes measurement of weight, height, blood pressure, heart rate, head circumstances, an inspection of dysmorphic features, and moto coordination are routinely assessed at start of treatment (T0) by a nurse specialist supervised by a child and adolescent psychiatrist as part of standard clinical care. A subset of the somatic screening (height, weight, waist circumference, blood pressure) is assessed at T1 and T3.
  Overweight and obesity prevalence for children are defined by the cut-off values on body mass index references and age according to the International Obesity Task Force [30]. In the United States and Europe, the diagnosis of persistent childhood hypertension is made when repeat blood pressure values on three separate visits are greater than the 95^th^ percentile for the age, gender, and height of the patient or ≥130/80 mmHg. The somatic measurements are assessed at T0 (Baseline), T1 (end of intervention), and T3 (12 months after baseline).
- H. Blood laboratory tests
  Blood samples (15 ml) are assessing glucose, HbA1C, total cholesterol, lipid profile, prolactin, vitamin D, thyroid stimulation hormone, and ALAT at T0 (Baseline) and for children with abnormalities during treatment , which are processed at local laboratory facilities. References from these facilities are used to interpret the results of the laboratory findings. For vitamin D, references from the American Academy of Pediatrics Committee on Nutrition and The Institute of Medicine are used corresponding to a serum 25-hydroxyvitamin D level of at least 50 nmol/liter (e.g., insufficiency <50-75 nmol/ml; deficiency <50nmol/l) [31–33].

#### Lifestyle measurements

A short screening questionnaire assessing sleep, physical activity, food consumption/habits, sleep, and screen time is used to differentiate a poor lifestyle from a normal lifestyle. All Lifestyle measures are assessed at T0 (Baseline), T1 (end of intervention), T2 (six months after baseline), T3 (12 months after baseline), except for the shuttle run test (not at T2 (six months after baseline)).

- I. Lifestyle parameter sleep: sleeping habits
  The Children’s sleep habit questionnaire (CSHQ) is a 33-item parent-report developed for children aged 4–10 [34], and has been used in youth ranging from 2–16 years old [35, 36]. Parents are asked to recall sleep behaviors occurring over a “typical” recent week [34–36]. Items are rated on a three-point scale: “usually” if the sleep behavior occurred five or more times/week; “sometimes” for two to four times/week; and “rarely” for zero to one [34–36]. There are eight scales: Bedtime resistance, Sleep onset delay, Sleep duration, Sleep anxiety, Night wakings, Parasomnias, Sleep-disordered breathing, and Daytime sleepiness [34–36]. The CSHQ can differentiate youth with sleep disorders from those without in typically developing and clinical populations [34, 37, 38]. A cutoff score of 44 is recommended for the 33-item version, where a higher score indicates a sleep disorder [39]. The Dutch CSHQ is the translated version of the American version. The reliability of the CSHQ seems to be adequate and the internal consistency is moderate [40].
- J. Lifestyle parameter sleep: sleep diary
  Parents are asked to fill in a visual sleep-wake diary to monitor the child’s sleeping habits and subjective sleep experience (regularity, total bedtime, total sleep time (TST), sleep onset latency (SOL), wake after sleep onset (WASO) and sleep efficiency (SE)).
- K. Lifestyle parameter Sleep: 24 h sleep/wake measurements and physical activity
  With actigraphy, a reliable non-invasive manner to monitor objective sleep parameters, the following parameters were collected: TST, SOL, WASO and SE [41] (http://www.actigraphcorp.com/products/wactisleep-bt-monitor/). Next to these sleep parameters, physical activity (ranging from sedentary behavior to light physical activity to moderate-to-vigorous intensity physical activity) and sedentary bouts (number of daily bouts of sedentary time lasting 1–4 minutes, 5–9 minutes, 10–14 minutes, 15–29 minutes, and 30+ minutes) are objectively and non-invasively measured as well. This way, children’s activity information can be measured in a natural setting for a continuous period. Children are asked to wear a wrist actigraph for a whole week.
- L. Lifestyle parameter Diet: Food consumption
  The child’s food consumption is measured through an online tool (‘Eetmeter’, Dutch Nutrition Centre) available at both the website of the Dutch Nutrition Centre and as a mobile app (free of charge). All products found in Dutch supermarkets are included in this application, including the standard quantities in which they are eaten. Users can enter all food consumed per main meal (breakfast, lunch, and dinner) and between-main meals (snacks). Parents are asked to track the child's food consumption for two weekdays and one weekend day.
- M. Lifestyle parameter diet: eating habits
  In addition to the food diary, the child’s eating habits are examined using sections B and C of the Primary Caregivers Questionnaire (PCQ) [42–44]. Parents are asked to complete 77 items assessing their child’s drinking and snacking behavior, including their attitude towards this.
- N. Lifestyle parameter physical activity: physical fitness
  Children’s physical fitness is measured using the 20-meter shuttle run test [45]. The 20-meter shuttle run test is valid for measuring cardiorespiratory fitness [46].
- O. Lifestyle parameter physical activity: physical activity questionnaire
  Furthermore, parents are asked to fill in a questionnaire, including questions about physical activity. Section D of the PCQ [42–44] is used to assess the physical activity behavior of the child. A total of 61 items assess their child's physical activity behavior in the past seven days, including their own attitude towards this.
- P. Lifestyle parameter screen time: screen activities
  Parents are asked to fill in a questionnaire on the screen activities of the child. Section E (sedentary behavior) of the PCQ [42–44] is used to assess the screen-viewing activities of the child. A total of 24 items assess their child’s screen time use and the amount of time spent on the screen, including their own attitude against this. Parents complete items assessing the screen viewing behavior of their child and themselves, including their own attitude towards this.

#### Measurements of costs

- Q. Used healthcare resources
  To examine the healthcare resources used by families and productivity losses (e.g., absence from work of parents) as a consequence of the child’s psychiatric disorder, the ‘Trimbos and iMTA questionnaire on Costs associated with Psychiatric illness’ (Tic-P questionnaire) [47] is used. The number of all healthcare contacts with a 3-month recall period is registered at T3 (12 months after baseline). With this data, the cost-effectiveness of the lifestyle intervention compared to CAU is calculated.

#### Other study parameters

- R. Parenting styles
  Parenting styles are examined using the Brief Scale of Parental Behavior (25 items) (BSBP: English translation of ‘Verkorte Schaal voor Ouderlijk Gedrag (VSOG)’) [48] at T0 (Baseline). This questionnaire is used to assess potential changes in pedagogical style during the intervention. The Brief Scale of Parental Behavior has a 5-point scale that ranges from 1 (*(almost) never*) to 5 (*(almost) always*) [48]. A low score on two subscales indicate inadequate parenting styles, while a high score on the three remaining subscales indicates inadequate parenting styles [48].
- S. Parenting functioning
  To assess changes in family functioning during treatment, the Family Functioning Questionnaire (28 items) (FFQ: English translation of ‘Vragenlijst Gezinsfunctioneren Ouders (VGFO)’) [49] is used at T0 (Baseline). The FFQ has a 4-point scale ranging from 1 (*not applicable*) to 4 (*completely applicable*), with lower scores suggesting more problems with family functioning [49].
- T. Prior beliefs
  To evaluate parents’ previous beliefs on the short-term and long-term efficacy and challenges of the lifestyle intervention, a 5-point scale questionnaire from 0 (*completely disagree*) to 5 (*completely agree*) is used. Prior beliefs are measured at T0 (baseline).
- U. Patient satisfaction
  Patient satisfaction and treatment compliance are measured using the GGZ-Jeugdthermometer [50]. This instrument permits institutions to overlook how clients and parents of clients appreciate the care provided. Parents assess their satisfaction with the information they received about the treatment, the team of psychologists, the amount of shared decision-making, and the results of the treatment. Furthermore, parents value their children's experiences during treatment and of their own. Finally, parents can rate their overall experience on a scale of 0 to 10. Patient satisfaction will be measured at T3 (12 months after baseline).

### Sample size and power

A sample size calculation using G-power 3.1 was conducted for the primary outcome measure, defined as the mean difference in the quality of life between two groups at three time points (baseline, 12 weeks intervention, 26 weeks follow-up). We used historical data on the Kidscreen-27 (a measure of QoL) from our own population to estimate the effect size. To detect a median effect size (Cohen’s f = 0.39) with a two-sided significance level of 5% and power of 80% with equal allocation to two arms would require 66 patients in the trial. To allow for 20% drop-out, 80 children are recruited in total.

### Data collection and management

Confidentiality is maintained throughout the current study. The handling of subjects’ personal data is in accordance with the European General Data Protection Regulation (in Dutch: Algemene Verordening Gegevensbescherming, AVG). To maintain anonymity of all data, participants will only be identifiable by a unique code assigned on the data of their inclusion. The code list will be digitally stored on the secured drive of Karakter, which is password protected, and is only accessible to researchers involved in the project. Non-anonymous data (e.g. informed consent documents), will be digitalized and stored in password-secured folders that provide restricted access. All local databases will be secured with password-protected access systems. The online electronic data capture software CASTOR EDC will be used to collect and store questionnaire data. Data collected during an assessment at the location, such as physical measurements, actigraph data, and outcomes of the WISC-V intelligence test, will also be entered and stored in CASTOR EDC. All paper documents are stored in a locker at Karakter Child and Adolescent Psychiatry. Access to this storage is accessible to only a select few researchers.

Parents will be reminded via e-mail (twice) and then via telephone by the researchers to reduce the chance of missing data. During the intake, researchers will explicitly mention 1-year participation to prevent drop-out at the long-term assessments (after 6 and 12 months). After completing these long-term data, families will also receive a small gift to make it more attractive to complete all four assessments. Parents can declare their travel expenses up to 20 euros. Children will receive a small gift after T0, T1, T2, and T3. Participants may withdraw from the study for any reason at any time.

### Statistical analysis

Data will be analyzed using SPSS V.29.0 for Windows (SPSS Incorporated). Descriptive statistics will be performed for baseline characteristics of the study population. Parametric data will be presented as means with SD, and non-parametric distributed variables as median with interquartile ranges (IQRs). A *p*-value < 0.05 will be considered statistically significant. To evaluate the impact of missing data on the outcome measurements, different approaches will be used, such as Multiple Imputation Strategy.

#### Primary study parameter(s)

Since the design is a repeated-measures RCT, repeated-measures techniques will be used to analyze the difference between the lifestyle intervention and CAU group for the quality of life. The repeated measures techniques will be based on the intention-to-treat principle, in which participants with at least one valid post-baseline follow-up are included. Analyses will be adjusted for the stratification factors (age group, sex). The study results will be reported in accordance with the Consolidated Standards of Reporting Trials (CONSORT) guidelines [51].

#### Secondary study parameter(s)

Concerning secondary outcome measurements, repeated measures techniques based on the intention-to-treat principle will be used to analyze the difference between the lifestyle intervention and the CAU group.

To get more insight into the prior beliefs of parents about short-term and long-term success, the outcomes of the 5-item questionnaire will be analyzed using the independent samples *t*-test to compare the lifestyle intervention group with the CAU group. Patient satisfaction and compliance with the intervention will be measured using the satisfaction measurement scale. The outcomes will be evaluated with a repeated-measures design.

The cost-effectiveness of the lifestyle intervention is measured with the TiC-P questionnaire. To assess this outcome, we will use an independent sample *t*-test to compare the lifestyle intervention with the CAU.

### Qualitative study

#### Procedures

For the qualitative inquiry part of this study, information will be obtained from a subsample of the quantitative study, encompassing a varied group of children, caregivers, and healthcare professionals involved to gain insight into their individual experiences and perspectives regarding the multi-modal lifestyle intervention. The qualitative part will consist of individual semi-structured in-depth interviews with children, their caregivers, and healthcare professionals. Caregivers will receive a formal letter explaining the qualitative study and an informed consent form. The researcher will only contact those with written informed consent to schedule an interview.

The researcher will assess all interviews. Interviews will preferably be face-to-face or via an online system (for example, Microsoft Teams) and will last approximately 45–60 min. Interviews will be digitally recorded or audio-recorded with the participant’s permission. The interviews will be recorded to ensure accuracy in data capture and transcribed verbatim. Immediately after each interview, participants’ non-verbal behaviors and emotions will be logged. Furthermore, a reflective journal will document the process of data collection. Caregivers and children will be interviewed separately. Each participant will be interviewed twice (before the start of the intervention and 26 weeks after the intervention).

The professionals will be interviewed at their workplace at a convenient time for them. A researcher will assess all interviews. Interviews will last 45–60 min. Each professional will be interviewed once (26 weeks after the intervention).

#### Power calculation

For the qualitative part of the study, participants from the intervention group will be included until data saturation is achieved. As such, power calculations are not applicable.

#### Data collection

The interview will consist of open questions to gain a comprehensive understanding of the participants' experiences [52]. We work with an iterative topic list. The topic list will be designed for exploratory purpose, with a focus on acquiring knowledge and experiences related to the topic. Possible questions for the children and their parents include: What are your expectations of the intervention? What should be maintained or strengthened in the intervention? What are the facilitators and barriers? What benefits have you derived from the intervention? How did you experience the intervention? What was your experience with the therapist? Etc.

The therapists will be interviewed to describe and evaluate therapeutic elements and implementation processes. Possible examples of questions are: What characterizes the referral procedure for the intervention? What are the barriers and facilitators for referral to the intervention? How do participants perceive the intervention offered? What aspects need to be added to your perspective? How do therapists experience the intervention implementation?

#### Qualitative analyses

ATLAS.ti version 9, a qualitative software package (Scientific Software Development GmbH, Berlin, Germany), is used to enter the transcripts of the interviews. The semi-structured interviews will be coded based on the principles of Grounded Theory [53], were a hierarchical process will be used (open coding, axial coding, and selective coding). After each interview, the interviewers will transcribe the recording verbatim and then code the transcript to reveal broad or initial categories or themes. The open codes that will be created aim to reflect the participants’ quotes as closely as possible, then axial and selective coding will follow. Throughout the analysis, researchers will use memos and reflective diaries to engage with the data and refine emerging themes through an iterative and inductive process. Data triangulation will be achieved by comparing interview data from professionals, children, and caregivers to explore the intervention from different perspectives. This will be done in a meeting with a large group of researchers by reflecting on their assumptions and discussing this within the team.

## Discussion

In this paper, the methodological design of the Movementss study is described. Furthermore, we explain how quantitative and qualitative data are combined in a mixed-methods approach. To our knowledge, this is the first RCT examining both short- and long-term effects on the effectiveness of an integrated lifestyle intervention program in children with various MI combined with CAU. It is estimated that the data collection will be completed in spring 2024 (*N* = 80) and that the follow-up data will be completed at the beginning of 2025.

We would like to emphasize two unique features and strengths of this study. First, the use of a mixed methods design to validate the quantitative and qualitative data findings. The purpose of pairing qualitative and quantitative components within this study is to better understand this intervention's tailored approach. Qualitative measurements can help to explain and interpret quantitative measurements; for example, the perception of caregivers and children on how they experienced the intervention can give a better insight into the ‘how’ and ‘why’ of its effectiveness. More importantly, it is crucial to explore both patients' and healthcare professionals' expectations and experiences of the intervention before implementing it in clinical practice or developing similar interventions in other settings.

Second, this intervention's systematic and tailored approach increases its transferability to clinical practice. From the literature, it is known that interventions will have a better chance of success when setting goals tailored to the needs, interests, and environment of the participants [19]. Because of the tailored approach used in this study, the translation to daily life will become more feasible, increasing the chances of a successful implementation at the end of the study.

In addition to the strengths of this study, we are aware of possible pitfalls. As tertiary care children are highly vulnerable, there is a possibility that this intensive intervention will be too demanding for the children and their parents, leading to dropouts. Also, as a result of the specific group of children in our study, we cannot generalize the results to the general population outside of tertiary care (i.e., primary and secondary care). However, if we find any positive effects in this population, it is likely that the intervention is effective in less specialized populations as well.

Overall, the awareness that lifestyle factors may be related to MI has grown rapidly, in both children and adolescent populations [3]. For instance, a meta-analysis concluded that short sleep duration was associated with a significantly increased risk of ADHD in children [54]. Furthermore, a recent lifestyle intervention study among children with autism described that interventions aimed at improving sleep, physical activity, and reducing screen time may have positive effects on the quality of life of these children [55]. Interventions in children, in particular, have great potential because of their preventive character for mental and physical outcomes. This is even more important in children with MI, since they are at high risk for developing chronic physical and mental symptoms (e.g., cardiovascular diseases, obesity, well-being, and quality of life) [56]. Therefore, this ongoing trial will investigate integrated lifestyle interventions in children with various MI, focusing on improving lifestyle habits and quality of life of young children with MI.

## Data Availability

The final dataset of the randomized controlled trial will be available (anonymized) from the corresponding author upon reasonable request at the end of the study.

## Abbreviations

MI: Mental illness
ADHD: Attention Deficit Hyperactivity Disorder
ASD: Autism Spectrum Disorder
RCT: Randomized Controlled Trial
CAU: Care As Usual
DSM-V: Diagnostic and Statistical Manual of Mental Disorders
WISC-V: Wechsler Intelligence Scale for Children – Fifth edition
CBCL: Child Behavior Checklist
CSHQ: Children’s Sleep Habits Questionnaire
FFQ: Family Functioning Questionnaire
VGFO: Vragenlijst Gezinsfunctioneren Ouders
TST: Total Sleep Time
WASO: Wake After Sleep Onset
PCQ: Primary Caregivers Questionnaire
Tic-P: Trimbos and iMTA questionnaire on Costs associated with Psychiatric illness
BSBP: Brief Scale of Parental
VSOG: Verkorte Schaal voor Ouderlijk Gedrag
AVG: Algemene Verordening Gegevensbescherming
SES: Social Economic Status
CBT: Cognitive Behavior Therapy
PMST: Psychomotor Systematic Therapy
IQ: Intelligence Quotient
